# Supplementary material for: HotSPOT: A Computational Tool to Design Targeted Sequencing Panels to Assess Early Photocarcinogenesis
Source: Cancers (Basel). 2023 Mar 5;15(5):1612. doi: 10.3390/cancers15051612 (PMC10001346; doi:10.3390/cancers15051612)
Supplement: Supplementary file 1 [file cancers-15-01612-s001.zip › cancers-2210466-supplementary.pdf]

Supplementary Table 1: Comparison of base pairs shared between 10,000 bp sequencing panel design of three datasets.

Dataset Comparison	Percent Overlap
Dataset A vs Dataset B	36.58%
Dataset A vs Dataset C	41.71%
Dataset B vs Dataset C	28.26%
Dataset A vs Dataset B vs Dataset C	20.42%

Supplementary Table 2: Example data table of .csv file format required for hotSPOT.

gene	chr	pos
ARID1A	1	27087486
FAT1	4	1.88E+08
MLL3	7	1.52E+08
TRIOBP	22	38151115
FGFR2	10	1.23E+08
SMAD4	18	48593495
CCND1	11	69456219
SALL1	16	51174418
CREBBP	16	3786799
NOTCH3	19	15300208
TP53	17	7578370
NF1	17	29587399
SMAD4	18	48593495
PTEN	10	89624297
TP53	17	7578176

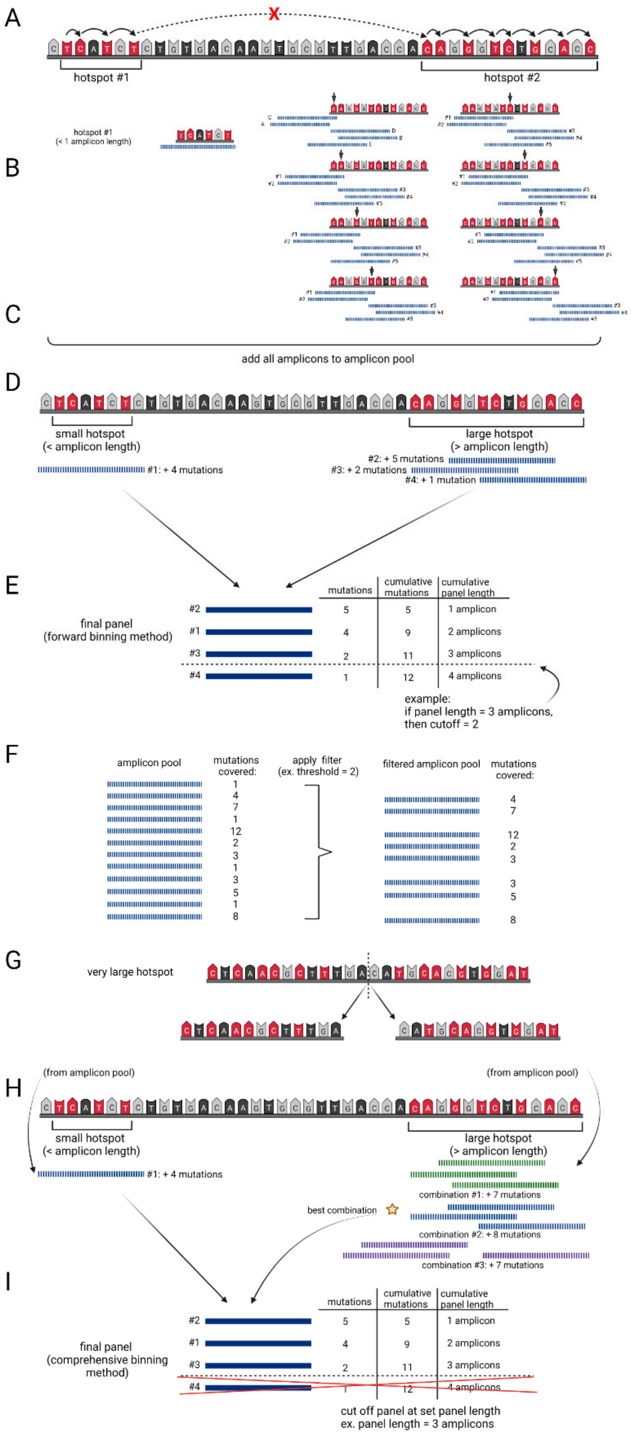

Supplementary Figure 1: Overview of hotSPOT forward and comprehensive binning algorithms

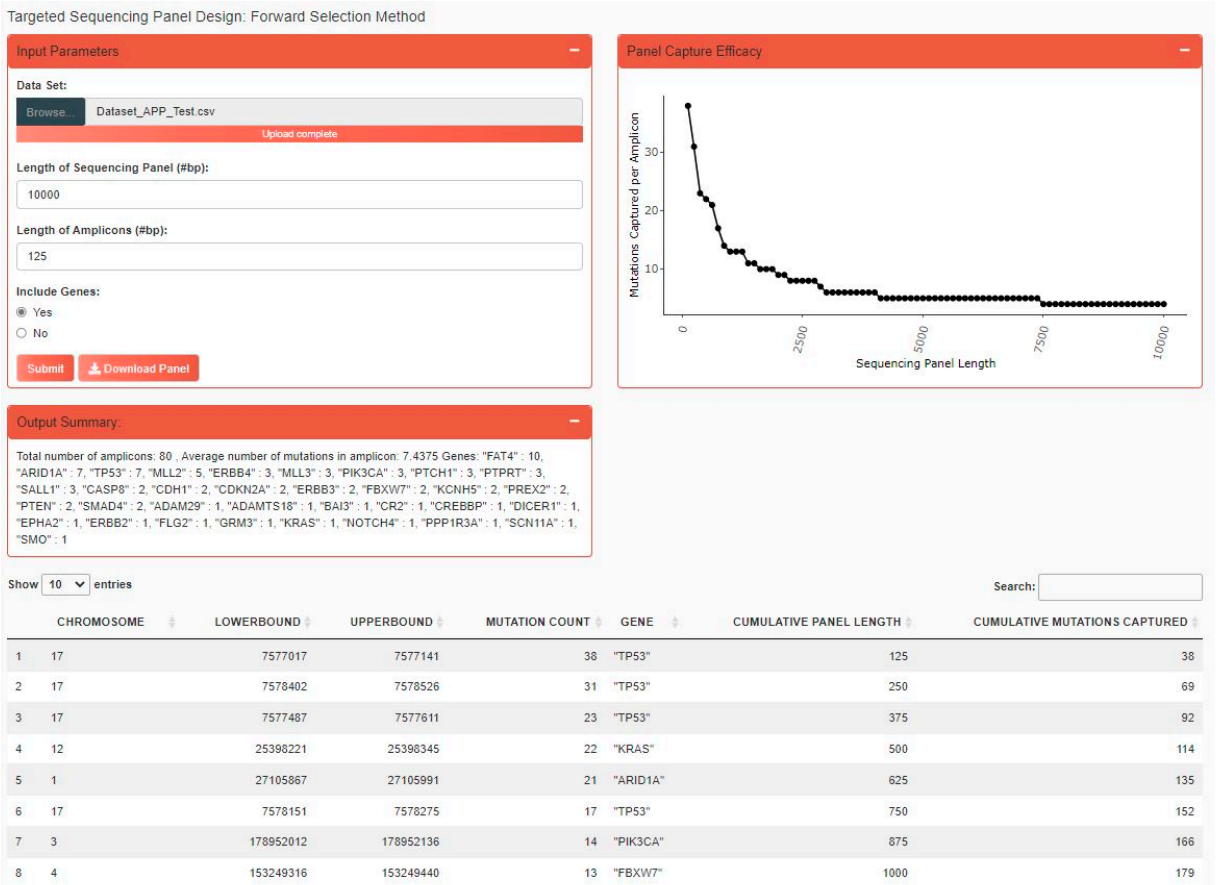

	CHROMOSOME	LOWERBOUND	UPPERBOUND	MUTATION COUNT	GENE	CUMULATIVE PANEL LENGTH	CUMULATIVE MUTATIONS CAPTURED
1	17	7577017	7577141	38	"TP53"	125	38
2	17	7578402	7578526	31	"TP53"	250	69
3	17	7577487	7577611	23	"TP53"	375	92
4	12	25398221	25398345	22	"KRAS"	500	114
5	1	27105867	27105991	21	"ARID1A"	625	135
6	17	7578151	7578275	17	"TP53"	750	152
7	3	178952012	178952136	14	"PIK3CA"	875	166
8	4	153249316	153249440	13	"FBXW7"	1000	179

Supplementary Figure 2: Visual representation of the forward algorithm hotSPOT R Shiny web application. User inputs .csv file of mutation dataset, desired length of sequencing panel and amplicon length. Application will output plot of panel capture efficacy, summary of mutation capture and genes included, and table of sequencing panel. Panel may be downloaded as .csv file.

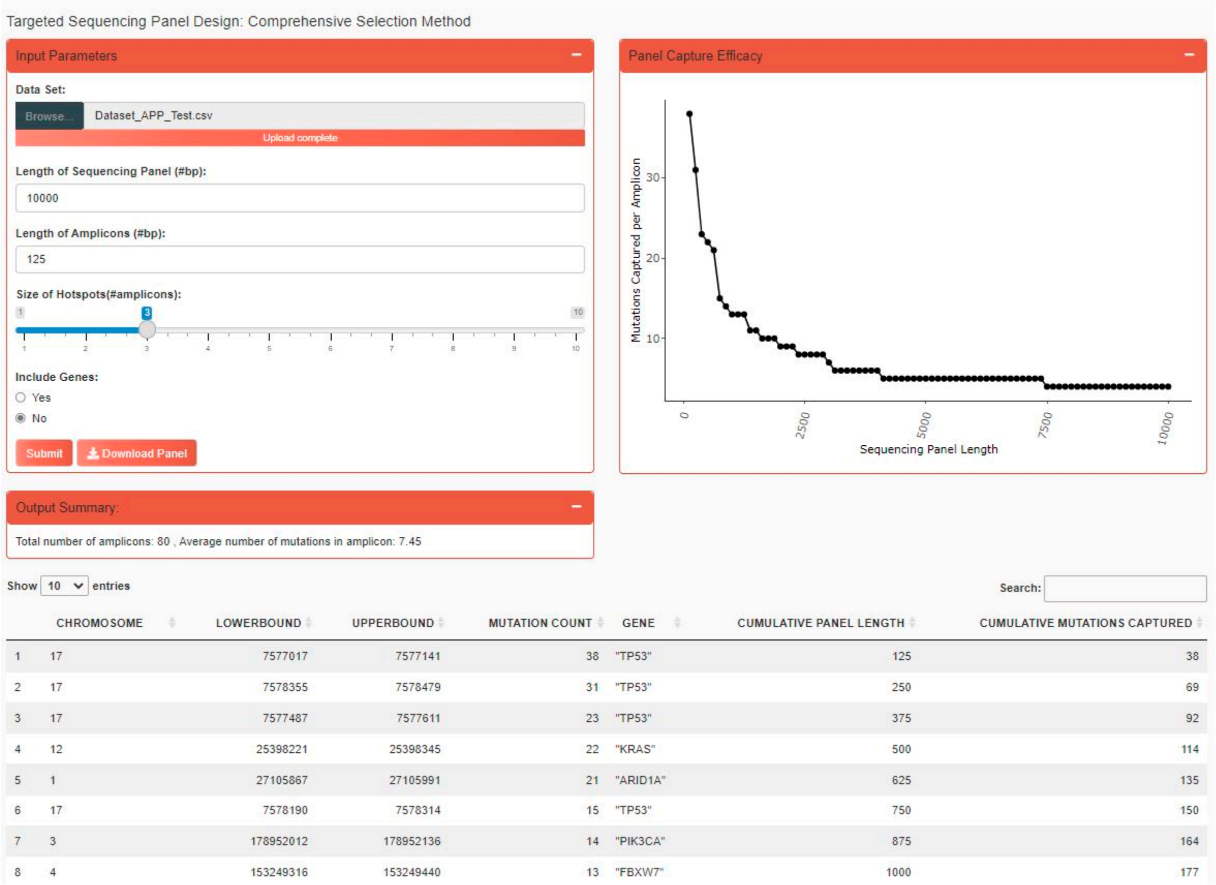

	CHROMOSOME	LOWERBOUND	UPPERBOUND	MUTATION COUNT	GENE	CUMULATIVE PANEL LENGTH	CUMULATIVE MUTATIONS CAPTURED
1	17	7577017	7577141	38	"TP53"	125	38
2	17	7578355	7578479	31	"TP53"	250	69
3	17	7577487	7577611	23	"TP53"	375	92
4	12	25398221	25398345	22	"KRAS"	500	114
5	1	27105867	27105991	21	"ARID1A"	625	135
6	17	7578190	7578314	15	"TP53"	750	150
7	3	178952012	178952136	14	"PIK3CA"	875	164
8	4	153249316	153249440	13	"FBXW7"	1000	177

Supplementary Figure 3: Visual representation of the comprehensive algorithm hotSPOT R Shiny web application. User inputs .csv file of mutation dataset, desired length of sequencing panel, amplicon length, and size of hotspots desired (Increasing hotspot size will increase capture efficacy, however also increase computation time). Application will output plot of panel capture efficacy, summary of mutation capture and genes included, and table of sequencing panel. Panel may be downloaded as .csv file.
